# Ureteroscopy Outcome and Its Determinants in a Resource-Limited Setting

**DOI:** 10.4314/ejhs.v32i5.10

**Published:** 2022-09

**Authors:** Seid Mohammed, Sultan Redi, Tekleberhan Berhe, Henok Teshome

**Affiliations:** 1 Department of Surgery, St. Paul's Hospital Millennium Medical College, Addis Ababa, Ethiopia

**Keywords:** Semirigid Ureteroscopy, Stone Clearance Rate, Success Rate, Complications

## Abstract

**Background:**

Ureteroscopy is a major diagnostic and therapeutic technique for lesions of the ureter and intrarenal collecting system.

**Methods:**

A retrospective chart review was done at St. Paul's Hospital Millennium Medical College, Ethiopia to determine the outcome of ureteroscopy and factors affecting it. The study period was from January 2018 to April 2018. Multivariate analysis was done to determine factors affecting stone clearance and success rate.

**Result:**

One hundred six patients who underwent semirigid ureteroscopy were included in the study. The male-to-female ratio was 1.8:1. The mean age of the patients was 36.4 years (±12.6). Ninety-six (90.6%) patients were found to have ureteric stones, while 9(8.5%) patients had a ureteric stricture. Ureteroscopy therapeutic interventions for stones were successful in 89 (92.7%) patients. The mean procedure time and postoperative hospital stay were 44 minutes (±23.7) and 2.5 days (±2.5) respectively. Intraoperative complications (ureteric avulsion, hemorrhage, and ureteral perforations) occurred in 6(5.7%) patients. The stone clearance rate was 54.7% (52). The site of obstruction was passed in 93 patients making the success rate of the procedure 87.7%. The absence of intraoperative complications was significantly associated with success rate. Patients with intraoperative complications have low success rate (20%) compared to patients without complications (92.3%), p=0.42.

**Conclusions:**

Semirigid ureteroscopy had a good success rate, especially for stones in the distal ureter and if there is no flexible ureteroscope, it is an acceptable alternative.

## Introduction

Ureteroscopy (URS) is retrograde instrumentation that involves inserting an endoscope into the ureter and calyceal system via the lower urinary tract. Although it was first described in 1912, it was in 1977 that Goodman reported the first rigid ureteroscopy for therapeutic purposes. It gradually became a standard method for diagnosing and treating ureter and intrarenal collecting system lesions. Urolithiasis, ureteric stricture, pelvic ureteric junction (PUJ) obstruction, ablation of transitional cell carcinoma, and retrieval of migrated stones are the most common indications for ureteroscopy (1).

Urinary stone disease is a major health issue that affects millions of people around the world, it affects 2–3% of the population and has a nearly 50% recurrence rate. Ureteric colic is a urological emergency due to the patient's excruciating pain. Many treatment modalities for ureteral calculi have been proposed in the literature, but with the recent development of small caliber semirigid and flexible deflectable ureteroscopes and a new generation of various lithotripters, ureteroscopic lithotripsy has become safer and more efficient (2).

Several authors have shown that ureteral strictures can be managed with dilatation or endoscopic incision using rigid and/or flexible ureteroscopes in retrograde, antegrade, or combined methods. The overall success rate was indicated to be around 60% (1).

URS can also be used as a diagnostic procedure. It can be used in the evaluation of ureteric transitional cell carcinoma, filling defects, and undiagnosed hematuria. Endourological approaches have been used to treat localized transitional cell carcinoma of the upper urinary tract in patients with contraindications to nephroureterectomy since the 1980s. Ureteroscopy performed to evaluate an upper urinary tract (UUT) filling defect greatly enhances diagnostic accuracy. It not only allows you to see the UUT, but it also allows you to biopsy any lesion you find for histopathological confirmation (1).

Ureteroscopy (URS) has evolved from a diagnostic procedure with limited visualization to an accurate surgical intervention allowing access to the entire urinary system since its first description over 30 years ago. However, as URS has become more popular, it has resulted in several incidents or complications, as well as new prevention strategies. Minor complications such as colic, fever, and hematuria to major complications such as ureteric perforation and avulsion may occur as a result of URS (2).

Although St. Paul's Hospital Millennium Medical College (SPHMMC) is one of the two leading referral hospitals in the capital, the endo-urology unit has not more than 2 years of experience in URS and uses a semirigid ureteroscope. The stones are fragmented with a pneumatic lithotripter. To the authors' knowledge, there is no similar study conducted at SPHMMC and there is only one study in the country. This study helps to know the outcome of URS and to suggest plans that improve it. The objective of the study was to determine the outcome of URS and the factors affecting it.

## Materials and Methods

A retrospective chart review was done at SPHMMC, Addis Ababa, Ethiopia. St. Paul's Hospital Millennium Medical College is a tertiary teaching hospital with 110 surgical beds. Department of Urology has 21 beds and one daycare endo urologic unit. Five senior urologists are working in the department and teach both undergraduate medical students and post-graduate urology and general surgery residents. All patients who underwent ureteroscopy from February 1, 2017, to January 31, 2018, G.C were included in the study. Patients who have undergone ureteroscopy in other healthcare facilities and were referred for repeated ureteroscopy, self-discharged patients, and patients with incomplete charts were omitted from the study.

The operating theatre logbook was used to identify patients and each patient's medical records and imaging were examined to extract the data. Urology residents who took training, collected data on patients' sociodemographic characteristics, diagnosis, site, and size of stones, complications, and stone clearance rate using a pretested data collection format. Data collection was monitored and data quality was verified daily. The data was analyzed using SPSS version 23. Binary logistic regression was used to determine the influence of various factors on stone clearance and success rate. Variables with p-values of < 0.3 during a bivariate analysis were further subjected to a multivariate analysis and considered significant at p-values < 0.05. Minor complications of ureteroscopy like fever and colic are not documented and is a limitation of the study.

This study was done based on the Helsinki declaration. An ethical approval letter was obtained from the SPHMMC Institutional Review Committee. Since the study is retrospective, the committee approved us to proceed without patient consent. The data collected was used solely for the study and patient information remained confidential.

In this study, stone clearance is when a patient has no residual stone in the ureter on imagining studies (KUB or non-contrast CT scan) during the first 6 months of follow-up after ureteroscopy. A successful procedure is when the ureteric obstruction is passed either with a double J stent or a ureteroscope.

## Results

Ureteroscopy was done for 115 patients during the study period. Nine patients were not included in the study because of incomplete data and lost charts, making the response rate 92.2%. Among the 106 patients, 68 (64.2%) were males and 38 (35.8%) were females with a 1.8:1 male to female ratio. The mean and median age of the patients were 36.4 years (±12.6) and 36.5 years (13–73 years) respectively. Most patients, 63 (59.4%), come from an urban area. Almost all, 104 (98.1%), patients were admitted as elective. Spinal anesthesia was used for 55(51.9%) patients, in the remaining 51(48.1%) patients the procedure was done under general anesthesia.

During ureteroscopy, 96 (90.6%) patients were found to have ureteric stones, while 9(8.5%) patients had a ureteric stricture and one patient had a normal ureteroscopy finding. Among the patients who had ureteric stones, 44 (45.8%) patients had left side stones and 45 (46.9%) patients had right ureteric stones. Seven (7.3%) patients had bilateral stones. Out of the 96 patients diagnosed with ureteric stone, the site of the stone was mentioned in 92 patients. Stones were found at the distal ureter in 38 (41.3%) patients, at the mid ureter in 13 (14.1%) patients, and in the proximal ureter in 41 (44.6%) patients. Among the 73 patients for whom the size of the stone was described, it was < 1 cm in 32 (43.8%) patients, 1–2 cm in 38 (52.1%) patients, and 2–3 cm in 3 (4.1%) patients. Thirty-seven (38.5%) patients who had ureteric stones had concomitant renal stones.

Ureteroscopy therapeutic interventions for stones were successful in 89 (92.7%) patients. For 36 (37.5%) patients only a double J stent was inserted, for 10 (10.4%) patients complete stone extraction was done, and for 41(42.7%) patients pneumonic or lesser stone fragmentation was done, in 2 (2.1%) patients the stone was dislodged proximally. In the remaining 7 (7.3%) patients with stone, ureteroscopy was only diagnostic, all therapeutic interventions were not successful (Table 1). For all patients with ureteric stricture, a stent was inserted except for one patient which had a severe stricture, so open resection and anastomosis was done.

**Table 1:** Ureteroscopic therapeutic procedures performed for ureteric stone at St. Paul's Hospital Millennium Medical College, Addis Ababa, Ethiopia, from February 1, 2017 to January 31, 2018

Procedures	Frequency	Percent
Double J stent insertion only	36	37.5
Stone extraction only	3	3.1
Stone extraction and double J stent insertion	7	7.3
Stone crushing only	4	4.2
Stone crushing and double J stent insertion	37	38.5
Proximal dislodging of stone and double J stent insertion	2	2.1
Failed procedure	7	7.3
Total	96	100

Out of the total 106 patients, a ureteric stent was inserted for 91(85.8%) patients, but for the remaining 15(14.2%) patients (1 stricture and 14 stone patients) it was not inserted. In patients with ureteric stones, the stent was inserted only for 82(85.4%) patients. The mean and median procedure times were 44 minutes (±23.7) and 40 minutes (10–120), respectively. The mean and median postoperative hospital stay was 2.5 days (±2.5) and 1 day (1–13).

Major intraoperative complications were documented in 6(5.7%) patients, all with ureteric stones. Ureteric avulsion occurred in 1 (0.9%) patient. Hemorrhage and Ureteral perforation occurred in 2 (1.9%) and 3 (2.8%) patients, respectively. The ureteric avulsions occurred during basket stone extraction. The patient was managed with Ureteroneocystostomy with Boary's flap. All perforations were associated with impaction of stone. One perforation was reconstructed by open procedure and for 2 of the perforations, URS was postponed, and repeat URS was successful. For both patients with gross hemorrhage, repeat URS was done some other time and was successful. All ureter perforations and hemorrhages occurred in patients with proximal ureter stones (p=0.006). No association was found between the size of the stone and complications (p=0.74). All above complications occurred in patients for whom stone crushing and JJ stent insertion was tried/done (p=0.4). The mean procedure time and postoperative hospital stay were 90 minutes (±50.6) and 5.7 days (±7.8) respectively, in patients with complications. This is nearly two times lower in patients without complications (2 days and 38.5 minutes).

Out of the 106 patients, 55(51.9%) patients were discharged improved without any other interventions. For 25 (23.6%) patients repeat URS was done. Eleven (10.4%) patients underwent ESWL, open surgical procedure was done for 10(9.4%) patients (3 were ureteric stricture). Three (2.8%) patients were referred for PCNL and for 2(1.9%) patients' other procedures were done.

Except one, all patients with ureteric stones had postoperative follow-up imaging. On postoperative imaging, stone was not visualized in 52 patients making the stone clearance rate 54.7%. In the remaining 43(45.3%) patients, the postoperative image showed remaining stones. Only 4(44.4%) patients with ureteric stricture had documented postoperative imaging, which showed normal position of the stent.

In both bivariate and multivariate analysis, the postoperative stone clearance rate was significantly associated with a distal ureteric location of stones (AOR=3.38; 95%CI 1.17–9.76; p=0.017). Patients with bilateral stones (28.6%), mid (46.2%) and proximal (43.9%) ureter stones, and large-sized (>1cm) stones had the lowest stone clearance rate even though it was not statistically significant (Table 2). The site of the obstruction was passed in 93 patients either through J stent insertion or proximal ureteroscopy evaluation, making the success rate of the procedure 87.7%. In 13 (12.3%) patients both proximal evaluation and J stent passage were not successful.

**Table 2:** Factors associated with stone clearance rate in patients who underwent ureteroscopy for ureteric stones at St. Paul's Hospital Millennium Medical Collège, Ethiopia, 2018

Factors		Complete No.(%)	Incomplete No.(%)	Crude Odds Ratio	Adjusted Odds Ratio
	Right	26(57.8)	19(42.2)	2.83(0.47–16.81)	
Stone Side	Left	24(54.5)	20(45.5)	3(0.5–17.74)	
	Bilateral	2(28.6)	5(71.4)	1	
	Distal Ureter	27(71)	11(29)	3.59*(1.3–10.2)	3.38*(1.17–9.76)
Stone	Mid Ureter	6(46.1)	7(53.9)	0.77(0.16–3.6)	
Location	Proximal Ureter	18(43.9)	23(56.1)	1	
	<1 cm	20(62.5)	12(37.5)	3.6(0.29–44.77)	
Stone Size	1–2 cm	16(42.1)	22(57.9)	1.5(0.12–17.46)	
	2–3 cm	1(33.3)	2(66.7)	1	
	Only JJ stent	17(47.2)	19(52.8)	-	-
	Stone extraction and JJ stent	9(90)	1(10)	-	-
Therapeutic	Stone Crushing and JJ stent	26(63.4)	15(36.6)	-	-
Procedures	Proximal dislodging and JJ stent	0	2(100)	-	-
	No procedure	0	6(100)	-	-

* Significantly associated at P-Value <0.05

On bivariate analysis, the presence of associated renal stone (p=0.001), proximal ureter location of the stones (p≤0.001), and the absence of intraoperative complications (p=0.002) were associated with success rate. However, on multivariate analysis, only the absence of intraoperative complications was significantly associated with success rate (AOR=19.61; 95%CI 1.50–255.98; p=0.023) (Table 3).

**Table 3:** Factors associated with ureteroscopic success rate in patients who underwent ureteroscopy for ureteric diseases at St. Paul's Hospital Millennium Medical Collège, Addis Ababa, Ethiopia, from February 1, 2017 to January 31, 2018

Factors		Successful No.(%)	Failed No.(%)	Crude Odds Ratio (95% CI)	Adjusted Odds Ratio (95% CI)
	Right	41(91.1)	4(8.9)	1.38(0.12–15.36)	
Stone Side	Left	38(86.4)	6(13.6)	0.64(0.07–6.25)	
	Bilateral	6(85.7)	1(14.3)	1	
Associated Renal	Yes	33(89.2)	4(10.8)	1*	
Stone	No	52(88.1)	7(11.9)	0.7(0.16–2.95)	
	Distal Ureter	37(97.4)	1(2.6)	5(0.55–45.48)	
Stone Location	Mid Ureter	8(61.5)	5(38.5)	0.22(0.04–1.09)	
	Proximal Ureter	36(87.8)	5(12.2)	1*	
Intra-operative	Yes	1(20)	4(80)	1	
complications	No	84(92.3)	7(7.7)	20.33*(3.06–135.03)	19.61*(1.5–255.9)
	<1 cm	29(90.6)	3(9.4)	-	-
Stone Size	1–2 cm	31(81.6)	7(18.4)	-	-
	2–3 cm	3(100)	0	-	-
Ureteroscope	Stone	85(88.5)	11(11.5)	-	-
finding	Stricture	7(77.8)	2(22.2)	-	-
	Normal	1(100)	0	-	-

* Significantly associated at P-Value <0.05

## Discussion

In this study, there was a male predominance (1.8 times) and the mean age of the patients was 36.4 years (±12.6). Other studies conducted in India, Ethiopia, and Egypt had also comparable findings (1–3). In our study, there was no discrepancy between clinical and ureteroscopic diagnosis, only one patient had a normal finding. In a study conducted by Andualem D. et.al. in Ethiopia, 20 (23.9%) patients had normal ureteroscopy (1).

Our study has found that only 14.1% of the ureteric stones were located at the mid ureter. This is slightly different from a study done in Pakistan by G.M. Subhani et.al, which found that almost half of the patients had a mid-ureteric stone (4).

Ureteroscopy therapeutic interventions for stones were successful in 89 (92.7%) patients. For 36 (37.5%) patients only double J stent was inserted, for 10 (10.4%) patients complete stone extraction was done and for 41(42.7%) patients stone fragmentation was done, in 2 (2.1%) patients the stone was dislodged proximally. In the remaining 7 patients with stones, the ureteroscopy was only diagnostic. However, the study from Ethiopia by D. Andualem et. al found complete stone extraction for 19 (50%) patients. Nine (23.7%) patients had only double J stenting while no therapeutic ureteroscopy procedure was done for 6 (15.8%) patients. For 2 (5.3%) patients the stone was crushed and in 2 (5.3%) others the stone was dislodged (1). Our study found that a stent was inserted for 85.4% of ureteric stone patients, similarly, Subhani et.al found that 78.81% had a stent (4). Saltzman B. recommended stenting patients following ureteroscopy when stones were big or fragmented into multiple pieces (5).

Our study found intraoperative complications in 5.7% of patients. Ureteric avulsion occurred in 1 patient. Hemorrhage and Ureteral perforation occurred in 2 and 3 patients respectively. Mugiya et al. in 2006 treated 54 patients with ureteric stones with 15.2 mm average diameter and found no complications. This absent complication can be due to the low sample size and the size of the stones was small (6). Subhani et.al and Alapont et al reported 1 and 3 ureteric avulsions (4,7). In the study by D. Andualem et.al, 1.2% of patients had ureteric perforation (1). We found that all perforations were associated with impaction of stone. Likewise, Tas et al. noted that ureteroscopic manipulation for impacted stones was associated with an increased incidence of perforation (11).

The stone clearance rate in our study was 54.7%. However, other studies conducted in Pakistan, India, and Egypt showed a much higher clearance rate (89%,100%, and 79% respectively) (1,3,4). This discrepancy can be explained by the lack of experience and advanced materials. Our endourology unit had not more than 2 years of experience in URS and we used semirigid ureteroscopy. The other reason is some of the above studies excluded patients with proximal stones which are relatively difficult to clear, but in our study majority, (58.7%) of patients had either mid or proximal stones. The fact that we do not have a holmium laser, which is demonstrated by Teichman et al, to be the best way to break up stones into the smallest fragments, can contribute to this low stone clearance (8).

Patients with bilateral stones, mid and proximal ureter stones, and large-sized (>1cm) stones had the lowest stone clearance rate. In both bivariate and multivariate analyses, the postoperative stone clearance rate was significantly associated with a distal ureteric location of stones. Similarly, the study conducted by Subhani GM et.al. found a low success rate for proximal ureter and >1 cm sized stones (4). Other studies by Ramello A. et.al and Matlaga BR. et.al have also found stone size and location as independent predictors of treatment failure (9,10). The highest stone clearance rate in our study was found in patients for whom basket stone extraction is done (90%). This was also supported by El-Qadhi M et.al., as grasping forceps have better stone clearance (99.2%) than pneumatic lithotripters (93.7%) (3).

Out of the 106 patients, 55(51.9%) patients were discharged improved without any other interventions. For 25 (23.6%) patients repeat URS was done, 11(10.4%) and 3(2.8%) patients were referred for ESWL and PCNL respectively, an open surgical procedure was done for 10(9.4%) patients and 2(1.9%) patients underwent other procedures. It is slightly different from a paper done in Ethiopia by D. Andualem et.al, of the 38 stone patients, 24 (63.2%) were discharged after ureteroscopy while 12 (31.5%) patients had to undergo other procedures and for 2 (5.3%) patients repeat ureteroscopy was done (2). This discrepancy in post-op outcomes and recommendations can be explained by a high rate of concomitant renal stones in our patients. In conclusion, semirigid ureteroscopy had a good success rate, especially for stones in the distal ureter and if there is no flexible ureteroscope, it is an acceptable alternative.
